# Risk Factors for Suboptimal Dialysis Initiation: A Prospective Cohort Study

**DOI:** 10.34067/KID.0000000895

**Published:** 2025-07-21

**Authors:** Amber O. Molnar, K. Scott Brimble, Sarah E. Bota, Yuguang Kang, J.P. Harmon, Swapnil Hiremath, Pierre A. Brown, Samuel A. Silver, Ayub Akbari

**Affiliations:** 1 Division of Nephrology, Department of Medicine, McMaster University, Hamilton, Ontario, Canada; 2 Department of Health Research Methods, Evidence, and Impact, McMaster University, Hamilton, Ontario, Canada; 3 ICES, Toronto, Ontario, Canada; 4 London Health Sciences Centre Research Institute, London, Ontario, Canada; 5 Northern Ontario School of Medicine, Sudbury, Ontario, Canada; 6 Division of Nephrology, Department of Medicine, University of Ottawa, Ottawa, Ontario, Canada; 7 Division of Nephrology, Department of Medicine, Queen's University, Kingston, Ontario, Canada

**Keywords:** chronic dialysis, chronic hemodialysis, CKD, chronic kidney disease, dialysis, ESKD, cohort studies

## Abstract

**Key Points:**

- Suboptimal dialysis initiation is common and is associated with increased morbidity and mortality.
- Lower hemoglobin and higher comorbidity were risk factors for suboptimal dialysis initiation, but health literacy and kidney disease knowledge were not.
- Modifiable patient risk factors for suboptimal dialysis initiation were not found. Our study highlights the complexity of preventing this outcome.

**Background:**

Suboptimal dialysis initiation is common and is associated with increased morbidity and mortality. We sought to determine risk factors for suboptimal dialysis initiation among patients with advanced CKD.

**Methods:**

This was a prospective cohort study that enrolled English-speaking patients without cognitive impairment followed in multidisciplinary kidney clinics across four regional kidney programs in Ontario, Canada. Patients completed a 6-month follow-up visit with further follow-up using health care administrative data. The primary outcome was suboptimal dialysis initiation defined by dialysis initiation with a central venous catheter, in patients younger than 75 years, or during a hospitalization. Adjusted cause-specific hazard models were used to examine the association of prespecified characteristics with suboptimal dialysis initiation.

**Results:**

Three hundred and sixty-six patients were included; 122 (33%) patients had a suboptimal dialysis start (69% of dialysis starts) over a median follow-up of 1.9 (interquartile range, 0.7–2.5) years. Higher hemoglobin (time varying) was associated with a lower risk of suboptimal dialysis initiation (adjusted hazard ratio, 0.96; 95% confidence interval [CI], 0.95 to 0.98). The mean (SD) hemoglobin in those with suboptimal dialysis initiation was 10.7 (1.5) g/dl. Higher comorbidity index and greater number of nephrologist visits within the past 6 months were associated with a higher risk of suboptimal dialysis initiation (adjusted hazard ratio, 1.17 [95% CI, 1.01 to 1.35] and 1.70 [95% CI, 1.39 to 2.08]; respectively). Measures of health literacy, kidney disease knowledge, and influenza vaccination were not associated with suboptimal dialysis initiation. A secondary analysis defining suboptimal dialysis initiation by dialysis initiation during a hospitalization showed similar results.

**Conclusions:**

Suboptimal dialysis initiation was common despite established nephrology follow-up. Our study did not find readily modifiable patient-related risk factors for suboptimal dialysis initiation.

## Visual Abstract

**Figure:**
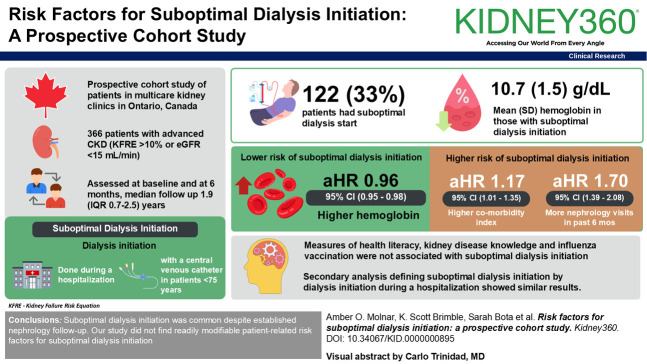

## Introduction

Suboptimal dialysis initiation, variously defined, but most often including the criteria of dialysis initiation during a hospitalization and/or with a central venous catheter (CVC),^1^ is associated with subsequent increased morbidity and mortality.^2–8^ As well, many patients with a suboptimal dialysis start are denied the opportunity to make an informed decision about their ESKD care.^9^ Early referral to a nephrologist is associated with a lower likelihood of suboptimal dialysis initiation.^5,10–14^ However, two previous Canadian studies reported that between 50% and 75% of patients with a suboptimal dialysis start were known to a nephrologist for over a year.^5,13,15^ Retrospective cohort and cross-sectional studies have reported various patient and system factors associated with suboptimal dialysis starts (*e.g*., increased age, lower serum albumin and hemoglobin at dialysis start, and lower number of nephrologist visits before dialysis).^2,4,10,11,13,15–20^ However, such studies are limited by selection biases (*i.e*., who gets referred to nephrology early) and confounding (*i.e*., AKI with no significant preexisting CKD). Reduced health literacy may also play a role, especially given the importance of education and shared decision making for KRT preparation.^9,21,22^ Studies examining health literacy in the US CKD population have found a high prevalence (25%) of low health literacy.^23^

We conducted a prospective cohort study to determine important risk factors for suboptimal dialysis initiation among patients with advanced CKD followed in multidisciplinary kidney care clinics in Ontario, Canada. As secondary aims, we examined the association of health literacy and kidney disease knowledge with hospitalizations, medication adherence, and patient-related delayed decision making with respect to KRT preparation, as these could be mediators of suboptimal dialysis initiation.

## Methods

### Design and Setting

We conducted a prospective cohort study in patients followed in multicare kidney clinics (MCKCs) across four regional kidney programs in the province of Ontario, Canada. MCKCs provide multidisciplinary care to adult patients (18 years or older) with advanced CKD defined by an estimated risk of kidney failure >10% over 2 years, as determined by the kidney failure risk equation, or an eGFR <15 ml/min per 1.73 m^2^.^24^ A nephrologist, nurse, dietitian, social worker, and pharmacist provide integrated care in the clinics. The focus of the clinics is to manage CKD complications, prevent CKD progression, and prepare patients for KRT (please see Supplemental Appendix for further details and individual center practices).^25^ Approval to conduct the study was obtained from the local Research Ethics Board. Patients enrolled in the study were assessed at baseline and at 6 months. Follow-up beyond 6 months was performed by linking prospectively collected study data with administrative health care databases held at ICES (ices.on.ca/) in Ontario, Canada. These datasets were linked using unique encoded identifiers and analyzed at ICES. The reporting of this study follows the REporting of studies Conducted using Observational Routinely-collected Data guidelines for observational studies (see Supplemental Table 1).^26^

### Study Population

Patients 18 years or older with advanced CKD who were followed in an MCKC and met MCKC eligibility criteria were included. Patients unable to provide informed consent, who had a documented history of dementia, who screened positive for dementia using the Mini-Cog, a 3 item test of cognitive function,^27^ and patients who only spoke French were excluded (the rapid estimate of adult literacy in medicine [REALM] test, which was used to assess health literacy, is not validated in French).^28–30^ The protocol specified that patients who were not fluent in English or French (the two official languages in Canada) could be included if they were able to provide informed consent, but they would not complete the REALM test, and it would be assumed that they had reduced health literacy because of communication challenges. However, no such patients were enrolled. Patients who were unable to read due to significant vision impairment (reading required for completion of the REALM test), and patients who could not be linked with ICES data were also excluded (see Supplemental Figure 1). Patients were recruited from April 24, 2019, to March 13, 2020, and were enrolled following informed consent.

### Patient Characteristics, Risk Factors, and Outcomes

Demographics, comorbidities, laboratory measurements, medications, and preventative health care (*e.g*., influenza vaccination) were determined at baseline using both primary data collection and ICES data. For patients who had not died or started KRT before 6 months, laboratory measurements, medications, and intercurrent hospitalizations were determined at a 6-month follow-up visit. KRT preparation (modality education and selection, dialysis access creation, and transplant assessment) was determined for all patients who completed a 6-month follow-up visit, including those who had started KRT. The number of nephrologist visits within the past 6 months and most recent hemoglobin were determined at baseline and throughout the follow-up using linked ICES databases (see Supplemental Table 2).

The REALM test was administered at the baseline visit; a score between 61 and 66 defines adequate health literacy.^30^ Either at baseline or at 6 months (if not yet on KRT), perceived kidney disease knowledge was evaluated using a previously validated nine-item questionnaire.^31^ Objective kidney disease knowledge was evaluated using a previously validated questionnaire.^32^

Outcomes are presented in Supplemental Table 2. The primary outcome was suboptimal dialysis initiation defined by dialysis initiation with a CVC, in patients younger than 75 years, or during a hospitalization (based on previous studies and expert consultation).^33^ Secondary outcomes included eGFR decline, patient-related delayed decision making with respect to KRT preparation, all-cause hospitalizations, emergency department visits, dialysis initiation, home dialysis within 90 days of dialysis initiation, and intended KRT modality being the same as the initiated modality. Medication adherence was determined at baseline using the validated Adherence to Refills and Medications Scale, which consists of 12 questions scored from 1 to 4;^34^ lower scores indicate better medication adherence. We examined acute illnesses associated with dialysis initiation using International Classification of Diseases, Tenth Revision diagnostic codes. Patient-related delayed decision making and intended KRT modality were determined using KRT preparation data obtained at 6 months. All other outcomes were determined using ICES databases with a maximum follow-up of March 31, 2022.

### Statistical Analysis

The representativeness of the study population was determined by comparing select baseline characteristics of the study population to the Ontario MCKC patient population (all patients with an MCKC visit registered in the Ontario Renal Reporting System between April 24, 2019, and March 13, 2020, and who met the MCKC eligibility criteria, *N*=14,045). Selected characteristics of the study population versus Ontario MCKC population and characteristics of patients who had a suboptimal dialysis start versus not (*i.e*., optimal dialysis initiation, preemptive kidney transplant, or no KRT initiation) were compared using one-way ANOVA and Kruskal-Wallis tests for continuous variables reported as mean and median, respectively, and chi-squared for binary and categorial variables. Crude frequency and incidence rate of suboptimal dialysis initiation were determined. Cause-specific hazard models were used to examine the association of prespecified characteristics with the outcome of suboptimal dialysis initiation versus not (*i.e*., optimal dialysis initiation, preemptive kidney transplant, or no KRT initiation). The following characteristics were included in the model: age (continuous), eGFR at the patient's first MCKC visit, body mass index (continuous), Charlson Comorbidity Index (CCI; continuous), hemoglobin (continuous, time varying covariate), health literacy (continuous score), influenza vaccination within the past year determined at the baseline visit (as a measure of preventative health care, y/n), and number of nephrologist visits over the past 6 months (0, 1, 2, 3, ≥4; time-varying covariate). Follow-up occurred until death, dialysis initiation, kidney transplant, or study end. Proportionality assumptions were verified by assessing Schoenfeld residuals. Prespecified sensitivity analyses were conducted using the following models: (*1*) the addition of urine albumin-to-creatinine ratio (ACR) at the first MCKC visit to the primary model (by 88.5 mg/g) and (*2*) the substitution of the REALM score with the objective and perceived kidney disease knowledge scores. The primary and sensitivity analyses were repeated using a prespecified alternate definition for suboptimal dialysis initiation (dialysis initiation during a hospitalization).

The mean eGFR decline was compared for patients with a suboptimal versus optimal dialysis initiation/preemptive kidney transplant versus no KRT initiation using a linear mixed-effects model with random, individual-specific intercepts to model eGFR versus time to get the distribution of eGFR change for each patient. The overall mean eGFR change in each outcome group was then compared. eGFR change was determined by taking all available serum creatinine measurements over each patient's follow-up time.

Crude frequencies and incidence rates or mean (SD) and median (interquartile range) were determined for all secondary outcomes. The following models were used to examine the association of health literacy and objective and perceived kidney disease knowledge with secondary outcomes: (*1*) subdistribution hazard models, accounting for the competing risk of death, were used for binary secondary outcomes; (*2*) generalized estimating equations were used for the outcomes of all-cause hospitalizations and emergency department visits; and (*3*) linear regression was used for the outcome of medication adherence. All models were adjusted for age, sex, educational level, household income, and CCI.

Missing values were imputed using hot deck imputation.^35^ Patients with missing kidney disease knowledge scores due to death or dialysis before 6 months (*n*=16) were excluded from models that included these scores. Two-sided *P* values < 0.05 were considered significant. All analyses were completed using SAS version 9.4 (SAS Institute, Cary, NC).

## Results

### Patient Characteristics

Study selection is presented in Supplemental Figure 1. A total of 1486 patients were screened, 1074 were deemed eligible, 384 consented, eight patients failed cognitive screening or withdrew consent, and ten patients could not be linked with ICES data. There were 366 patients included in the final study cohort. Compared with patients from the general MCKC population, study cohort patients were younger (67 versus 79 years), less likely to be female (33% versus 40%), had a lower eGFR (16.0 versus 17.8 ml/min per 1.73 m^2^), and higher urine ACR (median 1018.4 versus 798.3 mg/g). Selected comorbidities were similar (Supplemental Table 3). The study cohort overall had adequate health literacy (mean [SD] REALM score 63.6 [4.1]).

### KRT Preparation

At 6 months, nearly all patients had completed KRT modality education (100% versus 99%; suboptimal dialysis versus not), and a greater proportion of patients in the suboptimal dialysis group had selected a KRT modality (81% versus 61%). Many patients who went on to have a suboptimal dialysis start selected preemptive kidney transplant as their first KRT choice (*n*=31). Overall, KRT preparation was common, including among those who had a suboptimal dialysis start (Table 1).

**Table 1:** KRT preparation at 6 months by suboptimal dialysis initiation versus not

KRT Preparation	Suboptimal Dialysis Initiation		*P* Value
	Yes *N*=122	No (Optimal Dialysis Initiation, Preemptive Kidney Transplant or no KRT Initiation) *N*=244	
Selected a KRT modality	99 (81)	149 (61)	<0.001
**KRT modality selected (first choice)** ^a^			
Preemptive kidney transplant	31 (31)	50 (34)	0.79
Home hemodialysis	10 (10)	15 (10)	
In-center hemodialysis	20 (20)	34 (23)	
PD	38 (38)	48 (32)	
**Dialysis access created/inserted** ^b^	35 (29)	43 (18)	<0.001
Type of dialysis access^b^			
*AVF*	10 (8)	20 (8)	0.006
*AVG*	0 (0)	0 (0)	
*PD catheter*	25 (21)	23 (9)	
Referred for transplant workup^c^	26 (84)	36 (72)	0.22
Transplant assessment completed^c^	14 (45)	19 (38)	0.74
AVF, AVG, or PD catheter inserted^c^	14 (45)	16 (32)	0.11

AVF, arterio-venous fistula; AVG, arterio-venous graft; PD, peritoneal dialysis.

a Denominator patients who selected a KRT modality.

b Arterio-venous fistula, arterio-venous graft, or peritoneal dialysis catheter.

c Denominator patients who selected preemptive kidney transplant.

### Suboptimal Dialysis

There were 177 (48%) patients who initiated dialysis and 11 (3%) patients who received a preemptive kidney transplant. There were 122 (33%) patients with a suboptimal dialysis start (69% of dialysis starts) over a median follow-up of 1.9 (interquartile range, 0.7–2.5) years, giving an incidence rate of 20.3 per 100 person-years (>95% of these events met the criterion of dialysis initiation during a hospitalization). Death occurred in 44 (12%) patients. Compared with patients without a suboptimal dialysis start, patients with a suboptimal dialysis start had a lower eGFR (14.5 versus 16.8), higher urine ACR (median 1488.5 versus 813.3 mg/g), higher 2-year risk of kidney failure (mean 53% versus 38%), lower hemoglobin (10.8 versus 11.3 g/dl), and lower serum albumin (3.6 versus 3.9 g/dl) and were more likely to have completed KRT modality education (48% versus 32%) at baseline. They were more likely to have diabetes and cardiovascular disease, be prescribed loop diuretics and erythropoiesis-stimulating agents (ESA), and had a higher serum creatinine (3.3 versus 3.1 mg/dl) and urine ACR (median 1510.6 versus 808.0 mg/g) at their first MCKC visit. Health literacy, CKD knowledge, medication adherence, and preventative health care measures were all similar (Table 2). Patient characteristics at 6 months are presented in Supplemental Table 4.

**Table 2:** Baseline characteristics by suboptimal dialysis initiation versus not^a^

Characteristic	Suboptimal Dialysis Initiation		*P* Value
	Yes *N*=122	No (Optimal Dialysis Initiation, Preemptive Kidney Transplant or No KRT Initiation) *N*=244	
Age	67 (13.1)	67 (15)	0.61
Female	32 (26)	87 (36)	0.07
**Income quintile**			
1 (lowest)	28 (23)	59 (24)	0.26
2	24 (20)	60 (25)	
3	21 (17)	51 (21)	
4	33 (27)	42 (17)	
5 (highest)	16 (13)	32 (13)	
Rural residence	19 (16)	35 (14)	0.76
Self-reported primary spoken language English	109 (89)	223 (91)	0.84
**Highest education level** ^b^			
<High school	31 (25)	49 (20)	0.68
High school	30 (25)	84 (34)	
College degree or trade school	25 (21)	50 (21)	
University degree	24 (20)	40 (16)	
Professional or doctoral degree	9 (7)	15 (6)	
**Current marital status** ^c^			
Married/common law	80 (66)	151 (62)	0.30
Widowed or preferred not to answer	7 (6)	31 (13)	
Separated or divorced	16 (13)	35 (14)	
Single, never married	19 (16)	27 (11)	
Duration of follow-up in MCKC (mo)	18.9 (24.7)	23.6 (27.2)	0.11
Serum creatinine (mg/dl)	4.2 (1.3)	3.6 (1.2)	<0.001
eGFR (ml/min per 1.73 m^2^)^d^	14.5 (5.3)	16.8 (6.2)	<0.001
Urine ACR (mg/g)^e^	1488.5 (551.3–2954.0)	813.3 (308.0–1619.5)	<0.001
KFRE 2-yr risk of kidney failure (%)	53 (25.7)	38 (21.9)	<0.001
Serum creatinine at first MCKC visit (mg/dl)	3.3 (1.0)	3.1 (0.9)	0.03
eGFR at first MCKC visit (ml/min per 1.73 m^2^)	18.5 (6.3)	19.7 (6.0)	0.08
Urine ACR at first MCKC visit (mg/g)^e^	1510.6 (533.6–3228.3)	808.0 (287.6–1645.1)	<0.001
Potassium (mEq/L)	4.7 (0.6)	4.7 (0.6)	0.86
Hemoglobin (g/dl)	10.7 (1.5)	11.3 (1.7)	0.002
Serum bicarbonate (mEq/L)	22.2 (3.6)	22.7 (3.3)	0.29
Calcium (mg/dl)	8.8 (0.8)	9.2 (0.4)	<0.001
Phosphate (mg/dl)	5.0 (0.9)	4.0 (0.9)	<0.001
Albumin (g/dl)	3.6 (0.6)	3.9 (0.5)	<0.001
PTH (pg/ml)^e^	253.7 (146.2–359.3)	176.3 (113.2–288.6)	<0.001
BMI (kg/m^2^)	30.6 (6.6)	30.5 (6.9)	0.88
Systolic BP	138 (21.3)	135 (17.7)	0.16
Signs of volume overload	59 (48)	86 (35)	0.05
**Primary cause of ESKD**			
Diabetes	71 (58)	98 (40)	0.01
Hypertension	10 (8)	32 (13)	
GN	13 (11)	45 (18)	
Other	17 (14)	51 (21)	
Unknown	11 (9)	18 (7)	
Diabetes	84 (69)	128 (53)	0.003
Hypertension	97 (80)	178 (73)	0.17
Peripheral vascular disease	12 (10)	6 (3)	0.002
Heart failure	18 (15)	20 (8)	0.05
Stroke or TIA	15 (12)	14 (6)	0.07
Coronary artery disease	43 (35)	53 (22)	0.01
COPD	8 (7)	16 (7)	1.00
CCI	3.2 (1.3)	3.0 (1.3)	0.30
ACEi or ARB	77 (39)	124 (51)	0.20
*β*-blocker	67 (55)	96 (39)	0.01
Statin	94 (77)	167 (68)	0.09
Aspirin	54 (44)	92 (38)	0.23
Loop diuretic	71 (58)	103 (42)	0.004
Thiazide diuretic	13 (11)	40 (16)	0.14
ESA	25 (21)	23 (9)	0.004
Phosphate binders	25 (21)	38 (16)	0.24
Potassium binders	<6^f^	<6^f^	0.75
Influenza vaccination within 1 yr	85 (70)	170 (70)	1.00
Family physician visit within the past 6 mo	104 (85)	195 (80)	0.14
No. of nephrologist visits within the past 6 mo	2.8 (1.8)	2.2 (1.0)	<0.001
Attended a KRT modality education session	59 (48)	79 (32)	0.003
Mini-cog score	5.0 (0.8)	5.1 (0.8)	0.20
**REALM score**	63.6 (4.4)	63.6 (3.9)	0.98
Missing	<6^f^	<6^f^	0.52
**ARMS score**	14.3 (2.7)	13.8 (2.6)	0.12
Missing	<6^f^	<6^f^	0.48
**Objective kidney disease knowledge score (percentage of questions correct)** ^g^	75.0% (12.0)	75.0% (11.0)	0.65
Missing	12 (10)	17 (7)	0.34
**Perceived kidney disease knowledge score** ^g^ ^,^ ^h^	2.6 (0.5)	2.6 (0.6)	0.35
Missing	11 (9)	15 (6)	0.31

ACEi, angiotensin-converting enzyme inhibitor; ACR, albumin-to-creatinine ratio; ARB, angiotensin receptor blocker; ARMS, adherence to refills and medications scale; BMI, body mass index; CCI, Charlson Comorbidity Index; COPD, chronic obstructive pulmonary disease; ESA, erythropoiesis-stimulating agent; IQR, interquartile range; KFRE, kidney failure risk equation; MCKC, multicare kidney clinic; PTH, parathyroid hormone; REALM, rapid estimate of adult literacy in medicine; TIA, transient ischemic attack.

a Continuous measurements are reported as mean (SD) unless otherwise specified. Categorial measurements reported as *n* (%).

b Nine patients preferred not to answer this question.

c Less than six patients preferred not to answer this question, so the categories were collapsed in accordance with ICES privacy policies.

d Calculated using the CKD Epidemiology Collaboration equation.

e Median (interquartile range).

f In accordance with ICES privacy policies, cell sizes ≤5 cannot be reported.

g Kidney disease knowledge questionnaires could be completed at baseline or at 6 months.

h Perceived kidney disease knowledge was evaluated using a previously validated nine-item questionnaire,^31^ where patients subjectively rated their knowledge for each item on a scale from 1 (“I don't know anything”) to 4 (“I know a lot”). The average of a patient's ratings for each of the nine items was calculated.

Patients with a higher hemoglobin had a significantly lower risk of suboptimal dialysis initiation (adjusted hazard ratio [aHR], 0.96; 95% confidence interval [CI], 0.95 to 0.98), while patients with a higher CCI and greater number of nephrologist visits within the past 6 months had a significantly higher risk (aHR, 1.17; 95% CI, 1.01 to 1.35 and 1.70; 95% CI, 1.39 to 2.08; respectively). In addition, higher urine ACR at first MCKC visit was a risk factor (aHR, 1.01; 95% CI, 1.00 to 1.01). Age, body mass index, influenza vaccination status, health literacy, and kidney disease knowledge were not associated with suboptimal dialysis (Table 3). Analyses defining suboptimal dialysis by dialysis initiation during a hospitalization showed similar results (Supplemental Table 5).

**Table 3:** Risk factors for suboptimal dialysis initiation^a^

Risk Factor	HR	95% CI	*P* Value
**Model A (primary model; *n*=366)**			
Age	0.99	0.97 to 1.00	0.05
eGFR at first MCKC visit	0.99	0.95 to 1.02	0.44
BMI	1.01	0.98 to 1.03	0.62
Hemoglobin^b^	0.96	0.95 to 0.98	<0.001
CCI	1.17	1.01 to 1.35	0.04
Influenza vaccination in the past yr	1.14	0.75 to 1.75	0.53
No. of nephrologist visits^b^^,^^c^	1.70	1.39 to 2.08	<0.001
REALM score	1.01	0.96 to 1.05	0.75
**Model B (*n*=366)**			
Age	0.99	0.97 to 1.00	0.06
eGFR at first MCKC visit	0.99	0.95 to 1.02	0.45
BMI	1.01	0.98 to 1.04	0.44
Hemoglobin^b^	0.97	0.95 to 0.98	<0.001
CCI	1.18	1.02 to 1.36	0.02
Influenza vaccination in the past year	1.06	0.69 to 1.63	0.80
No. of nephrologist visits^b^^,^^c^	1.70	1.39 to 2.08	<0.001
REALM score	1.00	0.96 to 1.05	0.82
Urine ACR at first MCKC visit (per 88.5 unit increase, mg/g)	1.01	1.00 to 1.01	<0.001
**Model C (*n*=350)** ^d^			
Age	0.99	0.97 to 1.00	0.06
eGFR at first MCKC visit	0.99	0.95 to 1.02	0.45
BMI	1.01	0.98 to 1.04	0.44
Hemoglobin^b^	0.96	0.95 to 0.97	<0.001
CCI	1.15	0.99 to 1.35	0.07
Influenza vaccination in the past yr	1.06	0.68 to 1.64	0.81
No. of nephrologist visits^b^^,^^c^	1.69	1.37 to 2.09	<0.001
Objective kidney disease knowledge score	1.00	0.98 to 1.02	0.90
Perceived kidney disease knowledge score	0.89	0.62 to 1.28	0.53

ACR, albumin-to-creatinine ratio; BMI, body mass index; CCI, Charlson Comorbidity Index; CI, confidence interval; HR, hazard ratio; MCKC, multicare kidney clinic; REALM, rapid estimate of adult literacy in medicine.

a Referent group: patients with optimal dialysis initiation, preemptive kidney transplant, or no KRT initiation.

b Time varying covariate.

c Visits were binned into 6 month periods and the number of visits within each quarter was counted and categorized as 0, 1, 2, 3, and ≥4.

d Patients with missing values for the kidney disease knowledge scores who started dialysis, received a kidney transplant, or died before 6 months were excluded from this model.

Patients with a suboptimal dialysis start had the highest rate of eGFR decline (−4.12; 95% CI, −4.96 to −3.28 ml/min 1.73 m^2^ per year), which was significantly greater than patients who died or did not start KRT (−1.20; 95% CI, −1.66 to −0.75 ml/min per 1.73 m^2^ per year) but not significantly greater than patients who started dialysis optimally or received a preemptive kidney transplant (−3.76; 95% CI, −4.77 to −2.76 ml/min per 1.73 m^2^ per year).

The four most common main diagnoses associated with dialysis starts during a hospitalization were AKI (10.3%), acute myocardial infarction (7.7%), heart failure (6.8%), and type 2 diabetes (6.8%).

### Secondary Outcomes

Descriptive statistics for secondary outcomes are presented in Supplemental Tables 6 and 7. Increased health literacy was associated with initiation of the patient's intended KRT modality (aHR, 1.18; 95% CI, 1.05 to 1.32). Health literacy and kidney disease knowledge were not associated with patient-related delayed decision making, dialysis initiation, or home dialysis initiation within 90 days (Table 4; unadjusted results Supplemental Table 8). Higher health literacy and perceived kidney disease knowledge were associated with a lower risk of emergency department visits (adjusted related risk 0.99 [95% CI, 0.97 to 1.00] and 0.80 [95% CI, 0.69 to 0.93], respectively; Supplemental Tables 9 and 10). Health literacy and kidney disease knowledge were not associated with medication adherence (Supplemental Table 11).

**Table 4:** Adjusted associations of health literacy and kidney disease knowledge with KRT-related secondary outcomes

Outcome	Health Literacy			Objective Kidney Disease Knowledge^a^			Perceived Kidney Disease Knowledge^a^		
	HR^b^	95% CI	*P* Value	HR^b^	95% CI	*P* Value	HR^b^	95% CI	*P* Value
Patient-related delayed decision making	1.03	0.97 to 1.10	0.31	0.99	0.97 to 1.01	0.45	0.75	0.46 to 1.24	0.26
Dialysis initiation	1.01	0.96 to 1.06	0.67	1.00	0.98 to 1.01	0.72	1.01	0.76 to 1.36	0.93
Home dialysis initiation within 90 d	1.01	0.93 to 1.09	0.88	1.01	0.98 to 1.03	0.64	1.24	0.75 to 2.04	0.41
Intended KRT modality is the same as initiated modality	1.26	1.10 to 1.43	<0.001	1.02	1.00 to 1.05	0.08	1.35	0.85 to 2.15	0.20

CI, confidence interval; HR, hazard ratio.

a Patients with missing values for the kidney disease knowledge scores who started dialysis, received a kidney transplant, or died before 6 months were excluded from this model (*n*=350).

b Adjusted for age, sex, educational level, household income, center, Charlson Comorbidity Index.

## Discussion

Suboptimal dialysis initiation was common in this cohort of patients with advanced CKD followed in multidisciplinary kidney clinics. Lower hemoglobin and higher comorbidity index were both significant risk factors for suboptimal dialysis initiation, while measures of health literacy, kidney disease knowledge, and influenza vaccination (a marker of preventative health care) were not. Relative to patients without a suboptimal dialysis start, patients with suboptimal dialysis starts had received more KRT preparation at 6 months. Consistent with this, a greater number of nephrologist visits in the past 6 months was the strongest risk factor for suboptimal dialysis. Our results provide important insights toward preventing suboptimal dialysis starts and its associated morbidity.

Our results suggest that a greater burden of cardiovascular disease and diabetes is associated with suboptimal dialysis starts. This may be explained by a greater risk of acute events related to these comorbidities, such as myocardial infarctions and heart failure exacerbations, that result in worsening of kidney function prompting urgent dialysis initiation. This is further supported by acute myocardial infarction, heart failure, and diabetes being among the top four diagnoses seen in hospitalizations associated with dialysis initiation. Issues with volume management and the use of loop diuretics were also more common in those with suboptimal dialysis starts. This result is consistent with previous studies demonstrating congestive heart failure to be a significant risk factor for suboptimal dialysis initiation.^1,8,16^ Also consistent with previous studies,^14^ we found more rapid eGFR decline in the suboptimal dialysis group, which could be due to both acute illnesses resulting in acute on chronic kidney injury and having more rapidly progressive kidney disease (*i.e*., higher proteinuria in the suboptimal dialysis group). Preventative health care measures, such as influenza vaccination, that would potentially reduce complications related to comorbidities and infections did not seem protective.

Increased age, shown to be associated with suboptimal dialysis starts in multiple previous studies, was not a risk factor.^1^ This may be explained by the restriction of our cohort to patients actively receiving multidisciplinary kidney care, which would eliminate the confounding factor of late nephrology referral.^36^ Health literacy was a novel aspect of this study, included because low health literacy is associated with poor outcomes, such as higher CVC use and reduced preemptive kidney transplant.^23,37,38^ However, unexpectedly, almost all enrolled individuals had adequate health literacy, limiting the ability to examine health literacy as a risk factor for suboptimal dialysis starts. Despite this, we found a positive association between health literacy and patients initiating their intended KRT modality and reduced emergency department visits. With respect to the high health literacy of the cohort, this may be explained by the fact that research participants are not necessarily representative of the general CKD population.^39,40^ We examined the generalizability of our cohort by comparing selected characteristics to the Ontario MCKC population. The study cohort was similar; however, health literacy and cognitive measures were not available for comparison. Previous studies examining health literacy may have inadvertently included individuals with cognitive impairment, thus inflating the prevalence of reduced health literacy.^23^ In this study, individuals with known cognitive impairment or who screened positive for cognitive impairment using a validated, sensitive tool were excluded to eliminate this as a confounding factor.^27^

Consistent with previous studies, lower hemoglobin was a significant risk factor for suboptimal dialysis start.^1^ Anemia is associated with an increased risk of cardiovascular complications, kidney failure, and mortality in patients with CKD.^41^ A direct causal mechanism between anemia and suboptimal dialysis initiation is possible, potentially mediated by an increased risk of cardiovascular complications, but lower hemoglobin may also be a marker of more advanced CKD and of patients who are more prone to acute illness from infection, ESA resistance, and more rapid decline in kidney function. It is important to note that the mean hemoglobin was within targets for CKD,^42^ but with more ESA use, in the suboptimal dialysis group.

More nephrology visits before dialysis initiation were previously found to reduce the risk of suboptimal dialysis initiation.^20^ Unexpectedly, the opposite was found in this study. This suggests that suboptimal dialysis starts are occurring despite ample, close follow-up, and KRT preparation. The previous finding from retrospective studies might be a spurious result due to underlying selection bias. Robust risk prediction tools to more accurately identify patients at risk for suboptimal dialysis starts in a timely manner may be helpful.^43^ KRT preparation for such patients may need to begin even sooner; improved access to expedient KRT preparation (*i.e*., quicker living donor transplant workup) may also help. The other important point supported by our findings and others is that many suboptimal dialysis starts may not be preventable or easily predictable.^44^ Many acute illnesses are not preventable and may lead to an unanticipated acute on chronic kidney injury requiring urgent dialysis.

The prospective nature of this study with multicenter recruitment, restriction to patients with advanced CKD followed in multidisciplinary kidney clinics and extended follow-up using administrative data are strengths, but there are important limitations. First, we excluded individuals with cognitive impairment, and no non-English speaking patients were recruited; therefore, we could not examine these characteristics as risk factors for suboptimal dialysis start. Second, our findings may not be applicable to jurisdictions that use different models of CKD care. Finally, the use of a more sensitive health literacy tool or a larger sample size may have detected a wider range of differences in health literacy. It is also possible that with a larger sample size, we may have detected an association between health literacy and suboptimal dialysis initiation. This is suggested by the fact that we found some signal of a protective effect of higher health literacy demonstrated by an association with a reduced risk of emergency department visits and a higher risk of initiation of intended KRT modality. However, it is also possible that many emergency department visits are due to reasons unrelated to kidney disease, and therefore, while higher health literacy may protect against emergency department visits, it may not protect against suboptimal dialysis starts. It is also important to note that higher health literacy was not associated with a reduced risk of all-cause hospitalizations, and most suboptimal dialysis starts occurred during a hospitalization.

In conclusion, we did not find easily modifiable patient-related risk factors for suboptimal dialysis initiation. Preparation for KRT is complex with many opportunities for the process to fail. The next best step is likely improved kidney failure risk prediction tools applicable to the advanced CKD population that use a timeline over which KRT preparation typically occurs (*i.e*., 6–12 months) combined with starting KRT preparation sooner in high-risk patients. Machine learning may prove a valuable tool.^43^ Robust models would first need to be derived and validated, followed by testing to assess clinical impact.

## Supplementary Material

SUPPLEMENTARY MATERIAL

## Data Availability

This study includes clinical experimentation and received Institutional Review Board or Ethics Committee approval. All patients provided written informed consent.

## Data Availability Statements

Partial restrictions to the data and/or materials apply. The dataset from this study is held securely in coded form at ICES. While legal data sharing agreements between ICES and data providers (*e.g*., health care organizations and government) prohibit ICES from making the dataset publicly available, access may be granted to those who meet pre-specified criteria for confidential access, available at www.ices.on.ca/DAS (email: das@ices.on.ca). The full dataset creation plan and underlying analytic code are available from the authors upon request, understanding that the computer programs may rely on coding templates or macros that are unique to ICES and are therefore either inaccessible or may require modification.

## Supplemental Material

MCKC practices

Supplemental Figure 1. Study cohort.

Supplemental Table 1. RECORD checklist.

Supplemental Table 2. Key concept definitions.

Supplemental Table 3. Baseline characteristics of the study cohort and general MCKC population.

Supplemental Table 4. Patient characteristics at the 6-month follow-up visit by suboptimal dialysis versus not.

Supplemental Table 5. Risk factors for suboptimal dialysis (alternate definition of dialysis started during a hospitalization)

Supplemental Table 6. Outcome rates for binary secondary outcomes.

Supplemental Table 7. Descriptive statistics for continuous or count secondary outcomes.

Supplemental Table 8. Unadjusted associations of health literacy and CKD knowledge with KRT-related secondary outcomes.

Supplemental Table 9. Unadjusted associations of health literacy and kidney disease knowledge with hospitalizations and emergency department visits.

Supplemental Table 10. Adjusted associations of health literacy and kidney disease knowledge with hospitalizations and emergency department visits.

Supplemental Table 11. Association of health literacy and kidney disease knowledge with medication adherence.

## References

[1] Molnar AO Hiremath S Brown PA Akbari A. Risk factors for unplanned and crash dialysis starts: a protocol for a systematic review and meta-analysis. Syst Rev. 2016;5(1):117. doi:10.1186/s13643-016-0297-2 27431915 PMC4950106

[2] Caskey FJ Wordsworth S Ben T, . Early referral and planned initiation of dialysis: what impact on quality of life? Nephrol Dial Transplant. 2003;18(7):1330–1338. doi:10.1093/ndt/gfg156 12808170

[3] Couchoud C Moranne O Frimat L Labeeuw M Allot V Stengel B. Associations between comorbidities, treatment choice and outcome in the elderly with end-stage renal disease. Nephrol Dial Transplant. 2007;22(11):3246–3254. doi:10.1093/ndt/gfm400 17616533

[4] Loos C Briançon S Frimat L Hanesse B Kessler M. Effect of end-stage renal disease on the quality of life of older patients. J Am Geriatr Soc. 2003;51(2):229–233. doi:10.1046/j.1532-5415.2003.51062.x 12558720

[5] Mendelssohn DC Curtis B Yeates K, . Suboptimal initiation of dialysis with and without early referral to a nephrologist. Nephrol Dial Transplant. 2011;26(9):2959–2965. doi:10.1093/ndt/gfq843 21282303

[6] Mendelssohn DC Malmberg C Hamandi B. An integrated review of “unplanned” dialysis initiation: reframing the terminology to “suboptimal” initiation. BMC Nephrol. 2009;10:22. doi:10.1186/1471-2369-10-22 19674452 PMC2735745

[7] Metcalfe W Khan IH Prescott GJ Simpson K MacLeod AM. Can we improve early mortality in patients receiving renal replacement therapy? Kidney Int. 2000;57(6):2539–2545. doi:10.1046/j.1523-1755.2000.00113.x 10844623

[8] Kitamura M Yamashita H Sugiyama S, . Unplanned hemodialysis initiation: a retrospective analysis of patient characteristics and prognosis in an emergency hospital. Ther Apher Dial. 2024;28(6):931–940. doi:10.1111/1744-9987.14181 38946143

[9] Amir N McCarthy HJ Tong A. A working partnership: a review of shared decision-making in nephrology. Nephrology (Carlton). 2021;26(11):851–857. doi:10.1111/nep.13902 34010487

[10] Buck J Baker R Cannaby AM Nicholson S Peters J Warwick G. Why do patients known to renal services still undergo urgent dialysis initiation? a cross-sectional survey. Nephrol Dial Transplant. 2007;22(11):3240–3245. doi:10.1093/ndt/gfm387 17616535

[11] Marrón B Ortiz A de Sequera P, . Impact of end-stage renal disease care in planned dialysis start and type of renal replacement therapy--a Spanish multicentre experience. Nephrol Dial Transplant. 2006;21(suppl 2):ii51–ii55. doi:10.1093/ndt/gfl191 16825262

[12] Holland DC Lam M. Suboptimal dialysis initiation in a retrospective cohort of predialysis patients--predictors of in-hospital dialysis initiation, catheter insertion and one-year mortality. Scand J Urol Nephrol. 2000;34(6):341–347. doi:10.1080/003655900455396 11195896

[13] Chiu K Alam A Iqbal S. Predictors of suboptimal and crash initiation of dialysis at two tertiary care centers. Hemodial Int. 2012;16(suppl 1):S39–S46. doi:10.1111/j.1542-4758.2012.00744.x 23036035

[14] Heaf J Heiro M Petersons A, . Suboptimal dialysis initiation is associated with comorbidities and uraemia progression rate but not with estimated glomerular filtration rate. Clin Kidney J. 2020;14(3):933–942. doi:10.1093/ckj/sfaa041 33777377 PMC7986329

[15] Hughes SA Mendelssohn JG Tobe SW McFarlane PA Mendelssohn DC. Factors associated with suboptimal initiation of dialysis despite early nephrologist referral. Nephrol Dial Transplant. 2013;28(2):392–397. doi:10.1093/ndt/gfs431 23222418

[16] Brown PA Akbari A Molnar AO, . Factors associated with unplanned dialysis starts in patients followed by nephrologists: a retropective cohort study. PLoS One. 2015;10(6):e0130080. doi:10.1371/journal.pone.0130080 26047510 PMC4457723

[17] Castellano I Gallego S Labrador PJ Gómez-Martino JR Covarsí A. The start of renal replacement therapy in a Spanish department. Nefrologia. 2006;26(4):445–451. PMID: 17058856 17058856

[18] Crews DC Jaar BG Plantinga LC Kassem HS Fink NE Powe NR. Inpatient hemodialysis initiation: reasons, risk factors and outcomes. Nephron Clin Pract. 2010;114(1):c19–c28. doi:10.1159/000245066 19816040 PMC2842162

[19] Roubicek C Brunet P Huiart L, . Timing of nephrology referral: influence on mortality and morbidity. Am J Kidney Dis. 2000;36(1):35–41. doi:10.1053/ajkd.2000.8241 10873869

[20] Singhal R Hux JE Alibhai SM Oliver MJ. Inadequate predialysis care and mortality after initiation of renal replacement therapy. Kidney Int. 2014;86(2):399–406. doi:10.1038/ki.2014.16 24552848

[21] Frazier R Levine S Porteny T, . Shared decision making among older adults with advanced CKD. Am J Kidney Dis. 2022;80(5):599–609. doi:10.1053/j.ajkd.2022.02.017 35351579

[22] Devraj R Gordon EJ. Health literacy and kidney disease: toward a new line of research. Am J Kidney Dis. 2009;53(5):884–889. doi:10.1053/j.ajkd.2008.12.028 19324481

[23] Taylor DM Fraser SDS Bradley JA, . A systematic review of the prevalence and associations of limited health literacy in CKD. Clin J Am Soc Nephrol. 2017;12(7):1070–1084. doi:10.2215/CJN.12921216 28487346 PMC5498363

[24] Tangri N Stevens LA Griffith J, . A predictive model for progression of chronic kidney disease to kidney failure. JAMA. 2011;305(15):1553–1559. doi:10.1001/jama.2011.451 21482743

[25] Ontario Renal Network. Multi-Care Kidney Clinic Best Practices. Ontario Health; 2022.

[26] Benchimol EI Smeeth L Guttmann A, . The REporting of studies conducted using observational Routinely-collected health data (RECORD) statement. PLoS Med. 2015;12(10):e1001885. doi:10.1371/journal.pmed.1001885 26440803 PMC4595218

[27] Borson S Scanlan JM Chen P Ganguli M. The mini-cog as a screen for dementia: validation in a population-based sample. J Am Geriatr Soc. 2003;51(10):1451–1454. doi:10.1046/j.1532-5415.2003.51465.x 14511167

[28] Davis TC Wolf MS Arnold CL, . Development and validation of the rapid estimate of adolescent literacy in medicine (REALM-Teen): a tool to screen adolescents for below-grade reading in health care settings. Pediatrics. 2006;118(6):e1707–e1714. doi:10.1542/peds.2006-1139 17142495

[29] Fraser SD Roderick PJ Casey M Taal MW Yuen HM Nutbeam D. Prevalence and associations of limited health literacy in chronic kidney disease: a systematic review. Nephrol Dial Transplant. 2013;28(1):129–137. doi:10.1093/ndt/gfs371 23222414

[30] Safeer RS Keenan J. Health literacy: the gap between physicians and patients. Am Fam Physician. 2005;72(3):463–468. PMID: 16100861 16100861

[31] Wright Nunes JA Wallston KA Eden SK Shintani AK Ikizler TA Cavanaugh KL. Associations among perceived and objective disease knowledge and satisfaction with physician communication in patients with chronic kidney disease. Kidney Int. 2011;80(12):1344–1351. doi:10.1038/ki.2011.240 21832984 PMC3712792

[32] Wright JA Wallston KA Elasy TA Ikizler TA Cavanaugh KL. Development and results of a kidney disease knowledge survey given to patients with CKD. Am J Kidney Dis. 2011;57(3):387–395. doi:10.1053/j.ajkd.2010.09.018 21168943 PMC3053083

[33] Hassan R Akbari A Brown PA Hiremath S Brimble KS Molnar AO. Risk factors for unplanned dialysis initiation: a systematic review of the literature. Can J Kidney Health Dis. 2019;6:2054358119831684. doi:10.1177/2054358119831684 30899532 PMC6419254

[34] Kripalani S Risser J Gatti ME Jacobson TA. Development and evaluation of the adherence to refills and medications scale (ARMS) among low-literacy patients with chronic disease. Value Health. 2009;12(1):118–123. doi:10.1111/j.1524-4733.2008.00400.x 19911444

[35] Andridge RR Little RJ. A review of hot deck imputation for survey non-response. Int Stat Rev. 2010;78(1):40–64. doi:10.1111/j.1751-5823.2010.00103.x 21743766 PMC3130338

[36] Navaneethan SD Aloudat S Singh S. A systematic review of patient and health system characteristics associated with late referral in chronic kidney disease. BMC Nephrol. 2008;9:3. doi:10.1186/1471-2369-9-3 18298850 PMC2291456

[37] Zavacka M Skoumalova I Geckova AM, . Does health literacy of hemodialyzed patients predict the type of their vascular access? a cross-sectional study on Slovak hemodialyzed population. Int J Environ Res Public Health. 2020;17(2):675. doi:10.3390/ijerph17020675 31968703 PMC7013526

[38] Mazarova A Hiremath S Sood MM, . Hemodialysis access choice: impact of health literacy. Health Lit Res Pract. 2017;1(3):e136–e144. doi:10.3928/24748307-20170711-01 31294259 PMC6607797

[39] Decker E Kendrick J. Research in the CKD clinic: highs and lows. Adv Chronic Kidney Dis. 2014;21(4):344–348. doi:10.1053/j.ackd.2014.02.012 24969385 PMC4074357

[40] Gouveia R Cruz VT Almeida L. Sociodemographic and psychological characteristics influencing patients' willingness to participate in clinical trials. BMJ Open Qual. 2022;11(4):e002044. doi:10.1136/bmjoq-2022-002044 PMC9628668 36316063

[41] Hain D Bednarski D Cahill M, . Iron-deficiency anemia in CKD: a narrative review for the kidney care team. Kidney Med. 2023;5(8):100677. doi:10.1016/j.xkme.2023.100677 37415621 PMC10319843

[42] Moist LM Troyanov S White CT, . Canadian society of nephrology commentary on the 2012 KDIGO clinical practice guideline for anemia in CKD. Am J Kidney Dis. 2013;62(5):860–873. doi:10.1053/j.ajkd.2013.08.001 24054466

[43] Klamrowski MM Klein R McCudden C, . Derivation and validation of a machine learning model for the prevention of unplanned dialysis. Clin J Am Soc Nephrol. 2024;19(9):1098–1108. doi:10.2215/CJN.0000000000000489 38787617 PMC11390024

[44] Arulkumaran N Navaratnarajah A Pillay C, . Causes and risk factors for acute dialysis initiation among patients with end-stage kidney disease-a large retrospective observational cohort study. Clin Kidney J. 2019;12(4):550–558. doi: 10.1093/ckj/sfy118 31384448 PMC6671523

