# Supplementary material for: Risk Factors for Suboptimal Dialysis Initiation: A Prospective Cohort Study
Source: Kidney360. 2025 Jul 21;6(12):2175–84. doi: 10.34067/KID.0000000895 (PMC12708410; doi:10.34067/KID.0000000895)
Supplement: SUPPLEMENTARY MATERIAL [file kidney360-6-2175-s001.pdf]

**Supplemental Material**

**APPENDIX**

**Multi-care Kidney Clinic practices**

**FIGURES**

**Supplemental Figure 1.** Study cohort

**TABLES**

**Supplemental Table 1.** RECORD checklist

**Supplemental Table 2.** Key concept definitions

**Supplemental Table 3.** Baseline characteristics of the study cohort and general MCKC population

**Supplemental Table 4.** Patient characteristics at the 6-month follow-up visit by suboptimal dialysis vs. not

**Supplemental Table 5.** Risk factors for suboptimal dialysis (alternate definition of dialysis started during a hospitalization)

**Supplemental Table 6.** Outcome rates for binary secondary outcomes

**Supplemental Table 7.** Descriptive statistics for continuous or count secondary outcomes

**Supplemental Table 8.** Unadjusted associations of health literacy and CKD knowledge with kidney replacement therapy-related secondary outcomes

**Supplemental Table 9.** Unadjusted associations of health literacy and kidney disease knowledge with hospitalizations and emergency room visits

**Supplemental Table 10.** Adjusted associations of health literacy and kidney disease knowledge with hospitalizations and emergency department visits

**Supplemental Table 11.** Association of health literacy and kidney disease knowledge with medication adherence

**Appendix. Multi-care Kidney Clinic Practices**

- With respect to referral for vascular access creation, the process was similar at all included centres. The decision was ultimately a shared decision between the individual nephrologist and patient; all centres were recommended to follow the guidance provided in the Multi-Care Kidney Clinics Best Practices document:https://www.ontariorenalnetwork.ca/en/kidney-care-resources/clinical-tools/advanced-kidney-disease/multi-care-kidney-clinic-best-practices.
- With respect to PD catheter insertion, the timing was a shared decision between the individual nephrologist and the patient. Centres had a mixture of interventional radiologists, nephrologists and surgeons inserting the catheters. All centres followed similar absolute and relative contraindications for PD, as recommended by guidelines.[1] One centre (Ottawa) inserted buried PD catheters.
- With respect to anemia management, for all centres, this was ultimately at the discretion of the individual nephrologist, generally KDIGO guidelines for anemia management were followed.[2]
- With respect to CKD-MBD, for all centres, this was ultimately at the discretion of the individual nephrologist, generally KDIGO guidelines were followed.[3]
- All patients from all included centres had access to similar prescription drug coverage through the Ontario Drug Benefit plan.

**References**

1. Haggerty S, Roth S, Walsh D*, et al.* Guidelines for laparoscopic peritoneal dialysis access surgery. Surgical endoscopy 2014;28(11):3016-45.
2. Drüeke TB, Parfrey PS. Summary of the KDIGO guideline on anemia and comment: reading between the (guide)line(s). Kidney international 2012;82(9):952-960.
3. KDIGO 2017 Clinical Practice Guideline Update for the Diagnosis, Evaluation, Prevention, and Treatment of Chronic Kidney Disease-Mineral and Bone Disorder (CKD-MBD). Kidney international supplements 2017;7(1):1-59.

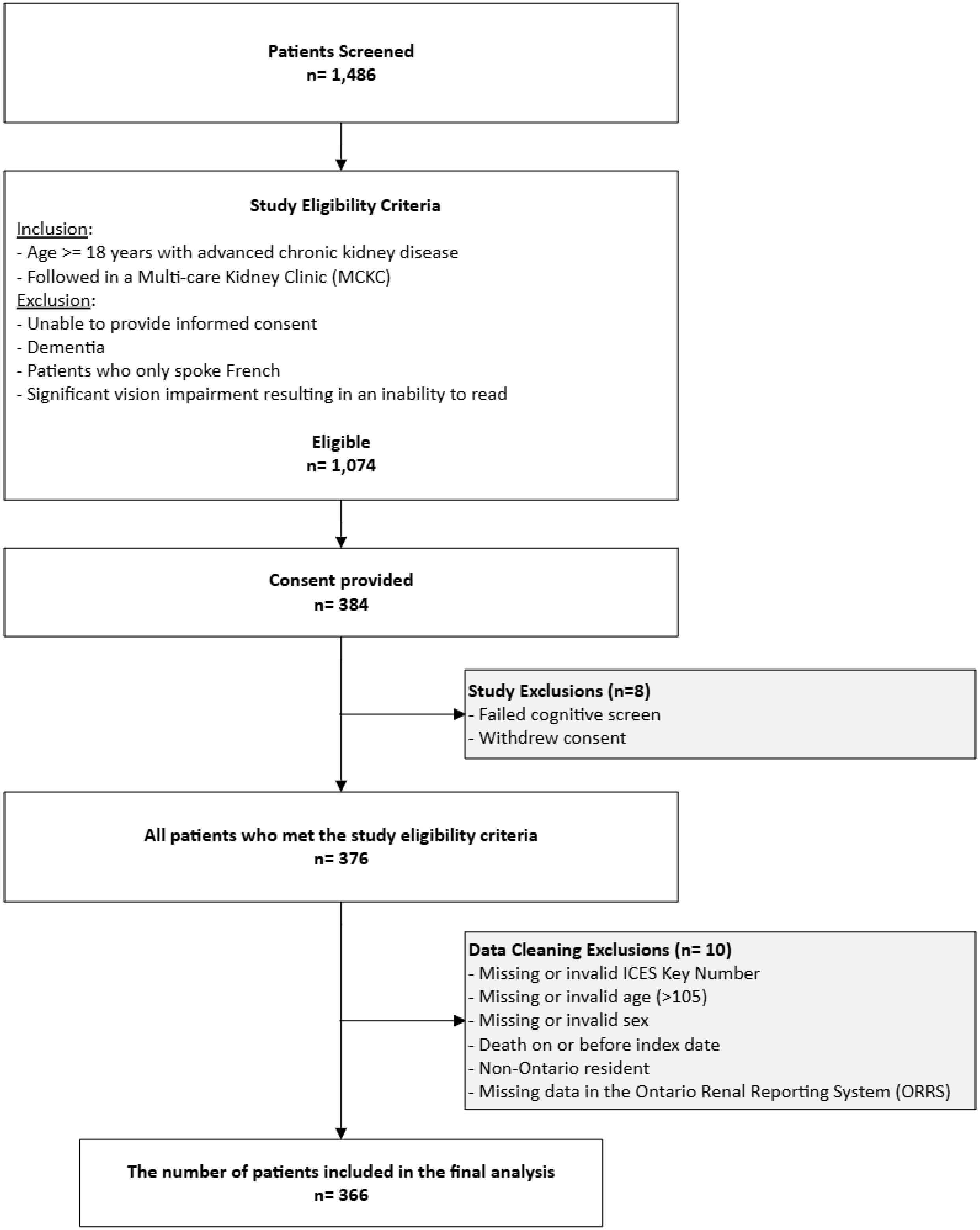


**Figure S1**. Study cohort

**Table S1.** Checklist of recommendations for reporting of observational studies using the REporting of studies Conducted using Observational Routinely-collected health Data (RECORD) Statement*

| | Item No | STROBE items | RECORD items | Reported |
|---|---|---|---|---|
| **Title and abstract** | 1 | (a) Indicate the study's design with a commonly used term in the title or the abstract.<br>(b) Provide in the abstract an informative and balanced summary of what was done and what was found. | (1.1) The type of data used should be specified in the title or abstract. When possible, the name of the databases used should be included.<br>(1.2) If applicable, the geographic region and time frame within which the study took place should be reported in the title or abstract.<br>(1.3) If linkage between databases was conducted for the study, this should be clearly stated in the title or abstract. | Abstract<br>Abstract<br>Abstract |
| **Introduction** | | | | |
| Background/ rationale | 2 | Explain the scientific background and rationale for the investigation being reported. | | Introduction |
| Objectives | 3 | State specific objectives, including any prespecified hypotheses. | | Introduction |
| **Methods** | | | | |
| Study design | 4 | Present key elements of study design early in the paper. | | Methods: Design and Setting |
| Setting | 5 | Describe the setting, locations, and relevant dates, including periods of recruitment, exposure, follow-up, and data collection. | | Methods |
| Participants | 6 | (a) Give the eligibility criteria, and the sources and | (6.1) The methods of study population selection (such as codes or algorithms used to identify | Methods |

| | | | | |
|---|---|---|---|---|
| | | methods of selection of participants. Describe methods of follow-up.<br>(b) For matched studies, give matching criteria and number of exposed and unexposed. | subjects) should be listed in detail. If this is not possible, an explanation should be provided.<br>(6.2) Any validation studies of the codes or algorithms used to select the population should be referenced. If validation was conducted for this study and not published elsewhere, detailed methods and results should be provided.<br>(6.3) If the study involved linkage of databases, consider use of a flow diagram or other graphical display to demonstrate the data linkage process, including the number of individuals with linked data at each stage. | Figure S1 |
| Variables | 7 | Clearly define all outcomes, exposures, predictors, potential confounders, and effect modifiers. Give diagnostic criteria, if applicable. | (7.1) A complete list of codes and algorithms used to classify exposures, outcomes, confounders, and effect modifiers should be provided. If these cannot be reported, an explanation should be provided. | Methods, Table S2 |
| Data sources/ measurement | 8 | For each variable of interest, give sources of data and details of methods of assessment (measurement). Describe comparability of assessment methods if there is more than one group. | | Methods, Table S2 |
| Bias | 9 | Describe any efforts to address potential sources of bias. | | Methods |
| Study size | 10 | Explain how the study size was arrived at. | | Methods, Figure S1 |
| Quantitative variables | 11 | Explain how quantitative variables were handled in the analyses. If applicable, | | Methods: Statistical Analysis |

| | | | | |
|---|---|---|---|---|
| | | describe which groupings were chosen and why. | | |
| Statistical methods | 12 | (a) Describe all statistical methods, including those used to control for confounding.<br>(b) Describe any methods used to examine subgroups and interactions.<br>(c) Explain how missing data were addressed.<br>(d) If applicable, explain how loss to follow-up was addressed.<br>(e) Describe any sensitivity analyses. | | Methods: Statistical Analysis |
| Data access and cleaning methods | | N/A | (12.1) Authors should describe the extent to which the investigators had access to the database population used to create the study population.<br><br>(12.2) Authors should provide information on the data cleaning methods used in the study. | Methods |
| Linkage | | N/A | (12.3) State whether the study included person-level, institutional-level, or other data linkage across two or more databases. The methods of linkage and methods of linkage quality evaluation should be provided. | Methods |
| **Results** | | | | |
| Participants | 13 | (a) Report numbers of individuals at each stage of study--e.g. numbers | (13.1) Describe in detail the selection of the persons included in the study (i.e., study population selection), including filtering based | Results, Figure S1 |

| | | | | |
|---|---|---|---|---|
| | | potentially eligible, examined for eligibility, confirmed eligible, included in the study, completing follow-up, and analyzed.<br>(b) Give reasons for non-participation at each stage.<br>(c) Consider use of a flow diagram. | on data quality, data availability, and linkage. The selection of included persons can be described in the text and/or by means of the study flow diagram. | |
| Descriptive data | 14 | (a) Give characteristics of study participants (e.g. demographic, clinical, social) and information on exposures and potential confounders.<br>(b) Indicate number of participants with missing data for each variable of interest.<br>(c) Summarize follow-up time (e.g. average and total amount). | | Results, Table 1, Table 2, Table S3, Table S4 |
| Outcome data | 15 | Report numbers of outcome events or summary measures over time. | | Results |
| Main results | 16 | (a) Give unadjusted estimates and, if applicable, confounder-adjusted estimates and their precision (e.g. 95% confidence interval). Make clear which confounders were adjusted for and why they were included. | | Results, Tables 3, 4, |

| | | | | |
|---|---|---|---|---|
| | | (b) Report category boundaries when continuous variables were categorized.<br><br>(c) If relevant, consider translating estimates of relative risk into absolute risk for a meaningful time period. | | |
| Other analyses | 17 | Report other analyses done (e.g. analyses of subgroups and interactions, and sensitivity analyses). | | Results, Tables S5-S11 |
| Key results | 18 | Summarize key results with reference to study objectives. | | Discussion |
| Limitations | 19 | Discuss limitations of the study, taking into account sources of potential bias or imprecision. Discuss both direction and magnitude of any potential bias. | (19.1) Discuss the implications of using data that were not created or collected to answer the specific research question(s). Include discussion of misclassification bias, unmeasured confounding, missing data, and changing eligibility over time, as they pertain to the study being reported. | Discussion |
| Interpretation | 20 | Give a cautious overall interpretation of results considering objectives, limitations, multiplicity of analyses, results from similar studies, and other relevant evidence. | | Discussion |
| Generalizability | 21 | Discuss the generalizability (external validity) of the study results. | | Discussion |
| **Other information** | | | | |

| | | | | |
|---|---|---|---|---|
| Funding | 22 | Give the source of funding and the role of the funders for the present study and, if applicable, for the original study on which the present article is based. | | Acknowledgment: Sources of Funding |
| Accessibility of protocol, raw data, and programming code | | N/A | (22.1) Authors should provide information on how to access any supplemental information such as the study protocol, raw data, or programming code. | Data Availability Statement |

*Reference: Benchimol EI, Smeeth L, Guttmann A, Harron K, Moher D, Petersen I, Sørensen HT, von Elm E, Langan SM, the RECORD Working Committee. The REporting of studies Conducted using Observational Routinely-collected health Data (RECORD) Statement. *PLoS Medicine* 2015 Oct 6;12(10):e1001885.

*Checklist is protected under Creative Commons Attribution (CC BY) license.

**Table S2.** Key concept definitions

| Concept | Databases | Definition |
|---|---|---|
| Nephrologist visits | Ontario Health Insurance Plan (OHIP)<br>Corporate Provider Database (CPDB) | Outpatient nephrologist visits<br><br>OHIP fee codes:<br>No restrictions<br>A161, A163, A164, A165, A166, A168, A865, C160, C161, C162, C163, C164, C165, C166, C167, C169, C865, W165, W160, W865, W166, W862, W864, W867, W869, W164, W162, W161, W163, W168<br><br>With OHIP or CPDB SPEC=16<br>A130, A131, A133, A134, A135, A136, A138, A435, C121, C122, C123, C124, C130, C131, C132, C133, C134, C135, C136, C137, C138, C139, C142, C143, C168, C435, C982, W121, W130, W131, W132, W133, W134, W138, W232, W234, W235, W236, W237, W239, W435, W972, W982<br><br>SERVDATE<br>SPEC=16<br><br>CPDB<br>SPEC= “16”<br>STARTSPEC<br>ENDSPEC<br>INDIC= Y |
| Hemoglobin | Suboptimal Dialysis Start Risk Factors (SODS-RF)<br>Ontario Laboratory Information System (OLIS) | hemo_fu1<br>hemo_rrti<br><br>Logical Observation Identifiers Names and Codes (LOINC)= 20509-6, 718-7 |

| | | |
|---|---|---|
| Kidney replacement therapy (KRT) modality education | Ontario Renal Reporting System (ORRS) | PATIENTTREATEMENTEVENTID_ENC<br>TREATMENTDATE<br><br>CLINICVISITID=1, 2<br>IDEDUCATIONPROVIDID=1<br>TREATMENTCHANGECD= VE, NP, VR |
| Sub-optimal dialysis initiation | OHIP<br>Discharge Abstract Database (DAD)<br>National Ambulatory Care Reporting System (NACRS)<br>Registered Person's Database (RPDB) | Defined as first evidence of dialysis that occurred either:<br><br>a. During a hospitalization or emergency department visit; OR<br>b. Using a central venous catheter (CVC) access in patients <75 years old.<br><br>All patients who do not meet either of the above criteria qualify as having 'optimal' dialysis initiation.<br><br>Dialysis<br>OHIP: R849, G323, G325, G326, G860, G862, G865, G863, G866, G330, G331, G333, G861, G082, G083, G085, G090, G091, G092, G093, G094, G095, G096, G294, G295, G864, H540, H740,<br>CCI: 1PZ21<br><br>Central Venous Catheter<br>OHIP: G324, G336, G327, G099, R848<br><br>SERVDATE<br>INDATE<br>ADMDATE<br>DDATE<br>REGDATE<br>DDATE<br>BDATE |

| | | |
|---|---|---|
| Alternate suboptimal dialysis definition | OHIP<br>DAD<br>NACRS<br>RPDB | Defined as first evidence of dialysis that occurred during a hospitalization or emergency department visit.<br><br>Dialysis<br>OHIP: R849, G323, G325, G326, G860, G862, G865, G863, G866, G330, G331, G333, G861, G082, G083, G085, G090, G091, G092, G093, G094, G095, G096, G294, G295, G864, H540, H740,<br>CCI: 1PZ21<br><br>SERVDATE<br>INDATE<br>ADMDATE<br>DDATE<br>REGDATE<br>DDATE<br>BDATE |
| Estimated glomerular filtration rate (eGFR) decline | OLIS | Inpatient and outpatient serum creatinine laboratory measurements converted to eGFR using the CKD-EPI (2009) equation.<br><br>LOINC= 14682-9 |
| All-cause hospitalizations | DAD | All instances of hospital admissions in the follow-up period.<br><br>ADMDATE |
| Emergency department visits | NACRS | All instances of emergency department visits in the follow-up period.<br><br>REGDATE |
| Patient related delayed decision-making | SODS-RF | Composite outcome where the patient qualifies if they meet any of the following variable combinations and answer types: |

| | | |
|---|---|---|
| | | 1. Modality education attended= “N” and reason why= “patient refusal” or “Other=’Missed’, ‘Refused’, ‘Could not be reached’, ‘Rescheduled’”*.<br><br>OR<br><br>2. Modality education attended = “Y” and selected a kidneyreplacement modality= “N”<br><br>OR<br><br>3. Has the participant been seen for a transplant assessment= “N” and reason why= missed appointment<br><br>OR<br><br>4. Participant received a referral for pre-emptive transplant= “N” and reason why = “patient refusal”<br><br>OR<br><br>5. Referral for access creation= “N” and reason why= “patient refusal”<br><br>OR<br><br>6. Has a dialysis access been created = “No” and reason why= “patient cancelled” or “patient missed appointment” |
| Medication adherence | SODS-RF | Adherence to Refills and Medications Scale (ARMS) |

| | | |
|---|---|---|
| | | Patient total score can range from 12 to 48, where a lower score= better adherence. Define more adherent as a score below the median.<br><br>arms1-12 |
| Dialysis initiation | OHIP<br>DAD<br>NACRS | OHIP: R849, G323, G325, G326, G860, G862, G865, G863, G866, G330, G331, G333, G861, G082, G083, G085, G090, G091, G092, G093, G094, G095, G096, G294, G295, G864, H540, H740,<br>CCI: 1PZ21 |
| Home dialysis initiation (Home HD or PD) within 90d | OHIP | Of those who initiated dialysis in the concept above, capture those who received dialysis at home (HD or PD)<br><br>OHIP: G865, G333, G864<br>SERVDATE<br>OUT_HOME |
| Intended (selected) KRT modality is the same as initiated KRT modality | SODS-RF<br>ORRS | In SODS-RF pull dial_mods= 2 (yes), no other condition needs to be met.<br><br>Then, pull the following concepts to determine the type of modality a patient selected prior to initiating dialysis: 'Patient selected a KRT modality', 'Modality selected' and 'Type of KRT'.<br><br>Then look in ORRS for the first evidence of dialysis initiation in follow-up. Pull modality ID associated with that record. Use the crosswalk in BC_MODSEL to convert ORRS modality to SODS modality.<br>Determine if KRT modality in ORRS and the modality selected in SODS-RF are equal.<br><br>SODS-RF<br>dial_mods |

| | | |
|---|---|---|
| | | ORRS<br>TREATMENTCHANGECD= N (New chronic registration), NA (New acute)<br>TREATMENTDATE<br>MODALITYID |
| Acute illnesses associated with dialysis initiation in hospital | SODS_RF<br>DAD<br>NACRS | Restricted to those who started dialysis in hospital (NACRS or DAD record, criteria A in suboptimal dialysis initiation concept).<br><br>Rank the reasons for DAD admissions (admitdx) and NACRS visits (main), 1-5, using the dx10codes. A single admission can contribute multiple admit dx dxcodes, include them all.<br><br>SODS-RF<br>rrti_hosp=2<br>rrti_hosr<br><br>DAD<br>ADMDATE<br>DXTYPE<br>ICD10<br><br>NACRS<br>REGDATE<br>REGTIME<br>dx10code1<br>ICD10 |

SODS-RF= primary data collection from chart at baseline and at 6 months

**Table S3. Baseline characteristics of the study cohort and general MCKC population[a]**

| Characteristic | Study cohort<br><br>N=366 | General MCKC population<br><br>N=14,045 | p value |
|---|---|---|---|
| Age | 67 (14.4) | 70 (14.1) | <0.001 |
| Female | 119 (32.5) | 5548 (39.5) | 0.007 |
| Income quintile | | | |
| 1 (lowest) | 87 (23.8) | 3567 (25.4) | 0.55 |
| 2 | 84 (23.0) | 3192 (22.7) | |
| 3 | 72 (19.7) | 2633 (18.7) | |
| 4 | 75 (20.5) | 2504 (17.8) | |
| 5 (highest) | 48 (13.1) | 2102 (15.0) | |
| Missing | 0 (0.0) | 47 (0.3) | |
| Rural residence | 54 (14.8) | 1543 (11.0) | 0.05 |
| Serum creatinine (µmol/L)[b] | 333.6 (114.2) | 303.1 (117.4) | <0.001 |
| eGFR (ml/min/1.73 $m^2$) | 16.0 (6.0) | 17.8 (6.9) | <0.001 |
| urine ACR (mg/mmol)[c] | 115.2 (43.4-224.9) | 90.3 (28.5-209.6) | 0.004 |
| Diabetes | 232 (63.4) | 9201 (65.5) | 0.40 |
| Hypertension | 321 (87.7) | 12,747 (90.8) | 0.05 |
| Charlson comorbidity index | 3.1 (1.3) | 3.2 (1.5) | 0.30 |
| Number of hospital admission in prior year | 0.6 (1.3) | 0.6 (1.1) | 0.88 |
| Number of nephrologist visits in prior 6 months | 2.9 (1.9) | 2.1 (1.3) | <0.001 |

[a]Continuous measurements are reported as mean (SD) unless otherwise specified. Categorial measurements reported as n (%).
[b]Conversion factor mg/dL= µmol/L*0.0113
[c]Median (IQR), conversion factor for mg/g= mg/mmol *8.84
Abbreviations: eGFR: estimated glomerular filtration rate, ACR: albumin to creatinine ratio

**Table S4. Patient characteristics at the 6-month follow-up visit by suboptimal dialysis initiation vs. not[a]**

| | Suboptimal dialysis initiation | | |
|---|---|---|---|
| **Characteristic** | **Yes N=116** | **No** (optimal dialysis initiation, pre-emptive kidney transplant or no KRT initiation) **N=229** | **p value** |
| Number of MCKC visits scheduled since baseline | 1.9 (1.3) | 1.6 (1.0) | 0.003 |
| Number of MCKC visits attended since baseline | 1.9 (1.3) | 1.5 (0.9) | 0.002 |
| Started KRT between the baseline and 6-month visits | 38 (32.8) | 14 (6.1) | <0.001 |
| Most recent serum creatinine (μmol/L)[b,c,d] | 380.3 (100.0) | 324.3 (108.6) | <0.001 |
| Most recent eGFR (ml/min/1.73 $m^2$)[b,c] | 13.6 (4.5) | 16.5 (5.8) | <0.001 |
| Most recent urine ACR (mg/mmol)[b,c,e] | 165.6 (73.8-273.8) | 86 (39.5-188.3) | <0.001 |
| KFRE 2-year risk of kidney failure (%)[b] | 54.9 (22.1) | 39.4 (22.5) | <0.001 |
| Potassium (mmol/L)[b,f] | 4.8 (0.6) | 4.9 (3.5) | 0.81 |
| Hemoglobin (g/L)[b,g] | 105.7 (14.2) | 115.1 (25.2) | 0.003 |
| Bicarbonate (mmol/L)[b,f] | 21.1 (3.5) | 22.2 (3.2) | 0.02 |
| Calcium (mmol/L)[b,h] | 2.2 (0.2) | 2.4 (1.5) | 0.34 |
| Phosphate (mmol/L)[b,i] | 1.5 (0.3) | 1.4 (0.3) | 0.002 |
| Albumin (g/L)[b,g] | 39.5 (5.2) | 40.8 (4.0) | 0.04 |
| PTH (pmol/L)[b,j] | 27.2 (18.4-39.3) | 22.2 (13.8-31.0) | 0.02 |
| ≥1 hospitalization since the baseline visit[b] | 14 (17.9) | 14 (6.5) | 0.003 |

[a]≤ 5 patients could not be contacted for the 6-month follow-up and 16 patients died prior to 6 months. These patients are excluded from the 6-month follow-up data. Continuous measurements are reported as mean (SD) unless otherwise specified. Categorial measurements reported as n (%).
[b]Excludes patients who started KRT prior to 6 months.
[c]3-month look-back.
[d]Conversion factor mg/dL= μmol/L*0.0113
[e]Median (IQR), conversion factor for mg/g= mg/mmol *8.84

[f]Conversion factor 1 mEq/L=1 mmol/L
[g]Conversion factor 1 g/dL= 1 g/L*0.1
[h]Conversion factor 1 mEq/L=1 mmol/L*2
[i]Conversion factor 1 mEq/L=1 mmol/L*3
[j]Median (IQR) conversion factor pg/mL= pmol/L*0.1061
Abbreviations: MCKC: multi-care kidney clinic, KRT: kidney replacement therapy, eGFR: estimated glomerular filtration rate, ACR: albumin to creatinine ratio, KFRE: kidney failure risk equation, PTH: parathyroid hormone

**Table S5. Risk factors for suboptimal dialysis initiation (alternate definition of dialysis started during a hospitalization)***

| **Risk factor** | **Hazard ratio** | **95% CI** | **p value** |
|---|---|---|---|
| **Model A (primary model) (n=366)** | | | |
| Age | 0.99 | 0.97-1.00 | 0.11 |
| eGFR at time of referral to MCKC | 0.99 | 0.95-1.02 | 0.45 |
| BMI | 1.01 | 0.98-1.04 | 0.42 |
| Hemoglobin[a] | 0.96 | 0.95-0.97 | <0.001 |
| Charlson comorbidity index | 1.15 | 0.99-1.34 | 0.06 |
| Influenza vaccination in the past year | 1.07 | 0.69-1.64 | 0.77 |
| Number of nephrologist visits[a,b] | 1.66 | 1.35-2.04 | <0.001 |
| REALM score | 1.00 | 0.96-1.05 | 0.91 |
| **Model B (n=366)** | | | |
| Age | 0.99 | 0.98-1.01 | 0.23 |
| eGFR at time of referral to MCKC | 0.98 | 0.95-1.02 | 0.37 |
| BMI | 1.02 | 0.99-1.04 | 0.25 |
| Hemoglobin[a] | 0.97 | 0.95-0.98 | <0.001 |
| Charlson comorbidity index | 1.17 | 1.01-1.35 | 0.04 |

| | | | |
|---|---|---|---|
| Influenza vaccination in the past year | 0.98 | 0.63-1.52 | 0.94 |
| Number of nephrologist visits[a,b] | 1.66 | 1.35-2.04 | <0.001 |
| REALM score | 1.00 | 0.96-1.04 | 0.98 |
| Urine ACR at first MCKC visit (per 10 unit increase, mg/mmol)[c] | 1.01 | 1.00-1.01 | <0.001 |
| **Model C (n=350)[d]** | | | |
| Age | 0.99 | 0.97-1.00 | 0.11 |
| eGFR at time of referral to MCKC | 0.99 | 0.95-1.02 | 0.45 |
| BMI | 1.02 | 0.99-1.04 | 0.25 |
| Hemoglobin[a] | 0.96 | 0.95-0.97 | <0.001 |
| Charlson comorbidity index | 1.14 | 0.97-1.35 | 0.10 |
| Influenza vaccination in the past year | 0.96 | 0.61-1.50 | 0.86 |
| Number of nephrologist visits[a,b] | 1.65 | 1.33-2.05 | <0.001 |
| Objective kidney disease knowledge score | 1.00 | 0.98-1.02 | 0.93 |
| Perceived kidney disease knowledge score | 0.85 | 0.58-1.23 | 0.39 |

[*]Referent group: patients with optimal dialysis initiation, pre-emptive kidney transplant or no KRT initiation

[a]Time varying covariate

[b]Visits were binned into 6 month periods and the number of visits within each quarter was counted and categorized as 0,1,2,3 and ≥4.

[c]Conversion factor for mg/g= mg/mmol *8.84

[d]Patients with missing values for the kidney disease knowledge scores who started dialysis, received a kidney transplant or died prior to 6 months were excluded from this model.

Abbreviations: eGFR: estimated glomerular filtration rate, MCKC: multi-care kidney clinic, BMI: body mass index.

**Table S6. Outcome rates for binary secondary outcomes**

| Outcome | N (%) | Total person years of follow up | Event rate per 100 person-years |
|---|---|---|---|
| Patient-related delayed decision making | 72 (19.7) | 153.8 | 46.8 |
| Dialysis initiation | 177 (48.4) | 599.9 | 29.5 |
| Home dialysis initiation within 90 days | 72 (19.7) | 606.7 | 11.9 |
| Intended modality is the same as initiated KRT modality | 66 (18.0) | 176.0 | 37.5 |

Abbreviation: KRT: kidney replacement therapy

**Table S7. Descriptive statistics for continuous or count secondary outcomes**

| Outcome | Mean (SD) |
|---|---|
| ARMS score (medication adherence) | 14.0 (2.6) |
| All cause hospitalizations | 0.9 (1.3) |
| Emergency department visits | 1.8 (2.4) |

**Table S8. Unadjusted associations of health literacy and kidney disease knowledge with kidney replacement therapy-related secondary outcomes**

| | Health literacy | | | Objective kidney disease knowledge[a] | | | Perceived kidney disease knowledge[a] | | |
|---|---|---|---|---|---|---|---|---|---|
| **Outcome** | **HR** | **95% CI** | **p value** | **HR** | **95% CI** | **p value** | **HR** | **95% CI** | **p value** |
| Patient-related delayed decision making | 1.03 | 0.97-1.10 | 0.31 | 0.98 | 0.96-1.00 | 0.04 | 0.75 | 0.50-1.11 | 0.15 |
| Dialysis initiation | 1.02 | 0.97-1.07 | 0.41 | 1.00 | 0.99-1.01 | 0.90 | 1.16 | 0.91-1.47 | 0.24 |
| Home dialysis initiation within 90 days | 1.05 | 0.98-1.12 | 0.18 | 1.01 | 0.99-1.03 | 0.40 | 1.30 | 0.87-1.95 | 0.20 |
| Intended KRT modality is the same as initiated modality | 1.18 | 1.07-1.30 | <0.001 | 1.01 | 0.99-1.04 | 0.24 | 1.45 | 0.95-2.21 | 0.08 |

[a]Patients with missing values for the kidney disease knowledge scores who started dialysis, received a kidney transplant or died prior to 6 months were excluded from this model (n=350).
KRT: kidney replacement therapy

**Table S9. Unadjusted associations of health literacy and kidney disease knowledge with hospitalizations and emergency department visits**

| | Health literacy | | | Objective kidney disease knowledge[a] | | | Perceived kidney disease knowledge[a] | | |
|---|---|---|---|---|---|---|---|---|---|
| **Outcome** | **RR** | **95% CI** | **p value** | **RR** | **95% CI** | **p value** | **RR** | **95% CI** | **p value** |
| All cause hospitalizations | 0.98 | 0.96-1.00 | 0.12 | 1.00 | 0.99-1.00 | 0.28 | 0.92 | 0.75-1.12 | 0.39 |
| All-cause emergency department visits | 0.98 | 0.97-0.99 | 0.002 | 0.99 | 0.99-1.00 | 0.005 | 0.80 | 0.70-0.91 | 0.001 |

[a]Patients with missing values for the kidney disease knowledge scores who started dialysis, received a kidney transplant or died prior to 6 months were excluded from this model (n=350).

**Table S10. Adjusted associations of health literacy and kidney disease knowledge with hospitalizations and emergency department visits**

| | Health literacy | | | Objective kidney disease knowledge[a] | | | Perceived kidney disease knowledge[a] | | |
|---|---|---|---|---|---|---|---|---|---|
| **Outcome** | **RR[b]** | **95% CI** | **p value** | **RR[b]** | **95% CI** | **p value** | **RR[b]** | **95% CI** | **p value** |
| All cause hospitalizations | 0.99 | 0.97-1.01 | 0.24 | 0.99 | 0.984-1.00 | 0.17 | 0.92 | 0.74-1.14 | 0.45 |
| All-cause emergency department visits | 0.98 | 0.97-1.00 | 0.002 | 0.993 | 0.99-1.00 | 0.002 | 0.80 | 0.69-0.92 | 0.003 |

[a]Patients with missing values for the kidney disease knowledge scores who started dialysis or received a kidney transplant or died prior to 6 months were excluded from this model (n=350).
[b]Adjusted for: age, sex, educational level, household income, centre, Charlson comorbidity index

**Table S11. Association of health literacy and kidney disease knowledge with medication adherence**

| | | Health literacy | | | Objective kidney disease knowledge[a] | | | Perceived kidney disease knowledge[a] | | |
|---|---|---|---|---|---|---|---|---|---|---|
| | | **Coefficient** | **95% CI** | **p value** | **Coefficient** | **95% CI** | **p value** | **Coefficient** | **95% CI** | **p value** |
| Medication adherence | Unadjusted | -0.05 | -0.12-0.02 | 0.18 | -0.02 | -0.04-0.01 | 0.66 | -0.17 | -0.65-0.31 | 0.50 |
| | Adjusted[b] | -0.02 | -0.09-0.05 | 0.51 | -0.01 | -0.03-0.01 | 0.45 | -0.50 | -0.98- -0.01 | 0.05 |

[a]Patients with missing values for the kidney disease knowledge scores who started dialysis or received a kidney transplant or died prior to 6 months were excluded from this model (n=350).
[b]Adjusted for: age, sex, educational level, household income, centre, Charlson comorbidity index
