# Supplementary material for: Risk Factors for Suboptimal Dialysis Initiation: A Prospective Cohort Study
Source: Kidney360. 2025 Jul 21;6(12):2175–84. doi: 10.34067/KID.0000000895 (PMC12708410; doi:10.34067/KID.0000000895)
Supplement: SUPPLEMENTARY MATERIAL [file kidney360-6-2175-s002.pdf]

ASN
American Society of Nephrology
JASN
Journal of the American Society of Nephrology
KIDNEY360
Accessing Our World From Every Angle
CJASN
Clinical Journal of the American Society of Nephrology

ASN Journal Disclosure Form

As per ASN journal policy, I have disclosed any financial relationships or commitments I have held in the past 36 months as included below. I have listed my Current Employer below to indicate there is a relationship requiring disclosure. If no relationship exists, my Current Employer is not listed.

A. Akbari reports the following:
Employer: University of Ottawa; Consultancy: Otsuka and AstraZeneca; Ownership Interest: Pfizer stocks; Merck Stocks; Research Funding: Otsuka and Astra Zeneca; Honoraria: AstraZeneca and Otsuka; Advisory or Leadership Role: AstraZeneca and Otsuka; and Speakers Bureau: AstraZeneca.

I understand that the information above will be published within the journal article, if accepted, and that failure to comply and/or to accurately and completely report the potential financial conflicts of interest could lead to the following: 1) Prior to publication, article rejection, or 2) Post-publication, sanctions ranging from, but not limited to, issuing a correction, reporting the inaccurate information to the authors' institution, banning authors from submitting work to ASN journals for varying lengths of time, and/or retraction of the published work.

Name: Ayub Akbari
Manuscript ID: K360-2025-000362R1
Manuscript Title: Risk factors for suboptimal dialysis initiation: a prospective cohort study
Date of Completion: May 27, 2025
Disclosure Updated Date: May 27, 2025

ASN
American Society of Nephrology
JASN
Journal of the American Society of Nephrology
KIDNEY360
Accessing Our World From Every Angle
CJASN
Clinical Journal of the American Society of Nephrology

ASN Journal Disclosure Form

As per ASN journal policy, I have disclosed any financial relationships or commitments I have held in the past 36 months as included below. I have listed my Current Employer below to indicate there is a relationship requiring disclosure. If no relationship exists, my Current Employer is not listed.

S. Bota reports the following:
Employer: London Health Sciences Centre

I understand that the information above will be published within the journal article, if accepted, and that failure to comply and/or to accurately and completely report the potential financial conflicts of interest could lead to the following: 1) Prior to publication, article rejection, or 2) Post-publication, sanctions ranging from, but not limited to, issuing a correction, reporting the inaccurate information to the authors' institution, banning authors from submitting work to ASN journals for varying lengths of time, and/or retraction of the published work.

Name: Sarah E. Bota
Manuscript ID: K360-2025-000362R1
Manuscript Title: Risk factors for suboptimal dialysis initiation: a prospective cohort study
Date of Completion: May 27, 2025
Disclosure Updated Date: March 14, 2025

ASN Journal Disclosure Form

As per ASN journal policy, I have disclosed any financial relationships or commitments I have held in the past 36 months as included below. I have listed my Current Employer below to indicate there is a relationship requiring disclosure. If no relationship exists, my Current Employer is not listed.

K. Brimble reports the following:
Employer: McMaster University; and Advisory or Leadership Role: Provincial Lead, Ontario Renal Network.

I understand that the information above will be published within the journal article, if accepted, and that failure to comply and/or to accurately and completely report the potential financial conflicts of interest could lead to the following: 1) Prior to publication, article rejection, or 2) Post-publication, sanctions ranging from, but not limited to, issuing a correction, reporting the inaccurate information to the authors' institution, banning authors from submitting work to ASN journals for varying lengths of time, and/or retraction of the published work.

Name: K. Scott Brimble
Manuscript ID: K360-2025-000362R1
Manuscript Title: Risk factors for suboptimal dialysis initiation: a prospective cohort study
Date of Completion: July 3, 2025
Disclosure Updated Date: July 3, 2025

ASN American Society of Nephrology | JASN Journal of the American Society of Nephrology | KIDNEY360 Accessing Our World From Every Angle | CJASN Clinical Journal of the American Society of Nephrology

ASN Journal Disclosure Form

As per ASN journal policy, I have disclosed any financial relationships or commitments I have held in the past 36 months as included below. I have listed my Current Employer below to indicate there is a relationship requiring disclosure. If no relationship exists, my Current Employer is not listed.

P. Brown reports the following:
Employer: The Ottawa Hospital; University of Ottawa; Consultancy: Otsuka Canada; Amgen Canada; Astra Zenaca Canada, GSK; Research Funding: Otsuka Canada; and Honoraria: Otsuka Canada; Astra Zenaca Canada.

I understand that the information above will be published within the journal article, if accepted, and that failure to comply and/or to accurately and completely report the potential financial conflicts of interest could lead to the following: 1) Prior to publication, article rejection, or 2) Post-publication, sanctions ranging from, but not limited to, issuing a correction, reporting the inaccurate information to the authors' institution, banning authors from submitting work to ASN journals for varying lengths of time, and/or retraction of the published work.

Name: Pierre-Antoine Brown
Manuscript ID: K360-2025-000362
Manuscript Title: Risk factors for suboptimal dialysis initiation: a prospective cohort study
Date of Completion: May 26, 2025
Disclosure Updated Date: May 26, 2025

ASN American Society of Nephrology
JASN Journal of the American Society of Nephrology
KIDNEY360 Accessing Our World From Every Angle
CJASN Clinical Journal of the American Society of Nephrology

ASN Journal Disclosure Form

As per ASN journal policy, I have disclosed any financial relationships or commitments I have held in the past 36 months as included below. I have listed my Current Employer below to indicate there is a relationship requiring disclosure. If no relationship exists, my Current Employer is not listed.

J. Harmon reports the following:
Employer: Health Sciences North Sudbury

I understand that the information above will be published within the journal article, if accepted, and that failure to comply and/or to accurately and completely report the potential financial conflicts of interest could lead to the following: 1) Prior to publication, article rejection, or 2) Post-publication, sanctions ranging from, but not limited to, issuing a correction, reporting the inaccurate information to the authors' institution, banning authors from submitting work to ASN journals for varying lengths of time, and/or retraction of the published work.

Name: John Paul Harmon
Manuscript ID: K360-2025-000362R1
Manuscript Title: Risk factors for suboptimal dialysis initiation: a prospective cohort study
Date of Completion: June 7, 2025
Disclosure Updated Date: June 7, 2025

ASN American Society of Nephrology | JASN Journal of the American Society of Nephrology | KIDNEY360 Accessing Our World From Every Angle | CJASN Clinical Journal of the American Society of Nephrology

ASN Journal Disclosure Form

As per ASN journal policy, I have disclosed any financial relationships or commitments I have held in the past 36 months as included below. I have listed my Current Employer below to indicate there is a relationship requiring disclosure. If no relationship exists, my Current Employer is not listed.

S. Hiremath reports the following:
Employer: University of Ottawa; Research Funding: Research Salary support from the Department of Medicine, University of Ottawa; and Advisory or Leadership Role: Editorial board: American Journal of Kidney Disease, Canadian Journal of Cardiology, American Journal of Hypertension; Board of Directors: NephJC (not for profit educational entity; unpaid volunteer position).

I understand that the information above will be published within the journal article, if accepted, and that failure to comply and/or to accurately and completely report the potential financial conflicts of interest could lead to the following: 1) Prior to publication, article rejection, or 2) Post-publication, sanctions ranging from, but not limited to, issuing a correction, reporting the inaccurate information to the authors' institution, banning authors from submitting work to ASN journals for varying lengths of time, and/or retraction of the published work.

Name: Swapnil Hiremath
Manuscript ID: #K360-2025-000362
Manuscript Title: Risk factors for suboptimal dialysis initiation: a prospective cohort study
Date of Completion: May 26, 2025
Disclosure Updated Date: May 26, 2025

ASN American Society of Nephrology | JASN Journal of the American Society of Nephrology | KIDNEY360 Accessing Our World From Every Angle | CJASN Clinical Journal of the American Society of Nephrology

ASN Journal Disclosure Form

As per ASN journal policy, I have disclosed any financial relationships or commitments I have held in the past 36 months as included below. I have listed my Current Employer below to indicate there is a relationship requiring disclosure. If no relationship exists, my Current Employer is not listed.

Y. Kang reports the following:
Employer: J.A. MacDonald London Limited

I understand that the information above will be published within the journal article, if accepted, and that failure to comply and/or to accurately and completely report the potential financial conflicts of interest could lead to the following: 1) Prior to publication, article rejection, or 2) Post-publication, sanctions ranging from, but not limited to, issuing a correction, reporting the inaccurate information to the authors' institution, banning authors from submitting work to ASN journals for varying lengths of time, and/or retraction of the published work.

Name: Yuguang Kang
Manuscript ID: K360-2025-000362
Manuscript Title: Risk factors for suboptimal dialysis initiation: a prospective cohort study
Date of Completion: May 26, 2025
Disclosure Updated Date: May 26, 2025

ASN American Society of Nephrology JASN Journal of the American Society of Nephrology KIDNEY360 Accessing Our World From Every Angle CJASN Clinical Journal of the American Society of Nephrology

ASN Journal Disclosure Form

As per ASN journal policy, I have disclosed any financial relationships or commitments I have held in the past 36 months as included below. I have listed my Current Employer below to indicate there is a relationship requiring disclosure. If no relationship exists, my Current Employer is not listed.

A. Molnar reports the following:
Employer: McMaster University; Advisory or Leadership Role: Canadian Society of Nephrology Board Member; and Other Interests or Relationships: Kidney Foundation of Canada; Canadian Institute of Health Research.

I understand that the information above will be published within the journal article, if accepted, and that failure to comply and/or to accurately and completely report the potential financial conflicts of interest could lead to the following: 1) Prior to publication, article rejection, or 2) Post-publication, sanctions ranging from, but not limited to, issuing a correction, reporting the inaccurate information to the authors' institution, banning authors from submitting work to ASN journals for varying lengths of time, and/or retraction of the published work.

Name: Amber O. Molnar
Manuscript ID: #K360-2025-000362
Manuscript Title: Risk factors for suboptimal dialysis initiation: a prospective cohort study
Date of Completion: May 23, 2025
Disclosure Updated Date: May 23, 2025

ASN
American Society of Nephrology
JASN
Journal of the American Society of Nephrology
KIDNEY360
Accessing Our World From Every Angle
CJASN
Clinical Journal of the American Society of Nephrology

ASN Journal Disclosure Form

As per ASN journal policy, I have disclosed any financial relationships or commitments I have held in the past 36 months as included below. I have listed my Current Employer below to indicate there is a relationship requiring disclosure. If no relationship exists, my Current Employer is not listed.

S. Silver reports the following:
Employer: Queen's University; Consultancy: Astra Zeneca; Research Funding: CSL Behring, Boehringer Ingelheim; Honoraria: Baxter, Otsuka, Novo Nordisk, Boehringer Ingelheim, Bayer; and Advisory or Leadership Role: Canadian Society of Nephrology Board Member.

I understand that the information above will be published within the journal article, if accepted, and that failure to comply and/or to accurately and completely report the potential financial conflicts of interest could lead to the following: 1) Prior to publication, article rejection, or 2) Post-publication, sanctions ranging from, but not limited to, issuing a correction, reporting the inaccurate information to the authors' institution, banning authors from submitting work to ASN journals for varying lengths of time, and/or retraction of the published work.

Name: Samuel A. Silver
Manuscript ID: K360-2025-000362
Manuscript Title: Risk factors for suboptimal dialysis initiation: a prospective cohort study
Date of Completion: May 23, 2025
Disclosure Updated Date: October 22, 2024
